# Efficacy and safety of oral anticoagulants in elderly patients with stable coronary artery disease and atrial fibrillation

**DOI:** 10.1186/s12959-022-00426-7

**Published:** 2022-10-31

**Authors:** Xu Zhang, Yangxun Wu, Chao Lv, Shizhao Zhang, Haiping Liu, Yuyan Wang, Yuting Zou, Liu’an Qin, Junmeng Zhang, Tong Yin

**Affiliations:** 1 Institute of Geriatrics, Beijing Key Laboratory of Aging and Geriatrics, National Clinical Research Center for Geriatric Diseases, Second Medical Center of Chinese PLA General Hospital, No.28 Fu Xing Road, Beijing, 100853 China; 2 Department of Cardiology, First Hospital of Tsinghua University, Beijing, China

**Keywords:** Stable coronary artery disease, Atrial fibrillation, Oral anticoagulants, Elderly, Antithrombotic treatment

## Abstract

**Background:**

This study aimed to evaluate the efficacy and safety of oral anticoagulants (OACs) in real-world elderly patients with comorbidities of stable coronary artery disease (SCAD) and atrial fibrillation (AF).

**Methods:**

Elderly patients (aged ≥ 65 years old) diagnosed with SCAD and AF were consecutively recruited and grouped into patients with or without oral anticoagulant (OAC) treatment. Follow-up was performed for 5 years. Major adverse cardiac events (MACEs) were defined as a composite of all-cause death, nonfatal myocardial infarction (MI), nonfatal stroke, and systemic embolism. Major bleeding outcomes were defined as events that were type ≥ 3 based on the Bleeding Academic Research Consortium (BARC) criteria. The net clinical outcomes were defined as the combination of MACEs and bleeding of BARC type ≥ 3.

**Results:**

A cohort of 832 eligible patients (78 ± 6.70 years) was included. Compared to the patients without OAC treatment (*n* = 531, 63.82%), the patients treated with OAC (*n* = 301, 36.18%) were much younger, had higher body mass index (BMI), and had lower prevalence of heart failure, chronic obstructive pulmonary disease (COPD), renal insufficiency, and previous myocardial infarction. During the follow-up of 5 years, compared to the patients without OAC treatment, patients with OAC had a significantly lower risk of MACEs (20.60% vs. 58.95%, adjusted HR: 0.21, 95% CI: 0.15–0.30, *p* < 0.001) but a higher risk of BARC ≥ 3 bleeding events (4.65% vs. 1.32%, adjusted HR: 4.71, 95% CI: 1.75–12.64, *p* = 0.002). In combination, a lower risk of net clinical outcomes could be observed in the patients with OACs (23.26% vs. 58.96%, adjusted HR: 0.27, 95% CI: 0.19–0.38, *p* < 0.001). Among the patients with OAC treatment, no significant difference was found for MACEs or BARC ≥ 3 bleeding events between the patients with or without comedications of oral antiplatelet agents.

**Conclusions:**

A net clinical benefit of efficacy and safety could be observed in OAC-treated elderly patients with SCAD and AF. This benefit is independent of the comedications of oral antiplatelet treatment.

## Introduction

Atrial fibrillation (AF), the most common cardiac arrhythmia, often coexists with stable coronary artery disease (SCAD), leading to high morbidity and mortality, especially in the elderly [1–4]. The pooled incidence of AF in patients with SCAD in a pairwise meta-analysis of 5 observational studies was 9.3% [5]. In a broad population of unselected ambulatory patients with SCAD, AF was presented as a frequent comorbidity with the incidence of 19% [6]. Elderly patients (age > 75 years) have the greatest mortality and morbidity risk attributable to SCAD, which is enriched by the high prevalence of comorbidities including AF [7]. However, despite its frequent occurrence in practice, there has been little evidence to guide therapy for comorbid chronic coronary artery disease (CAD) and AF in elderly individuals. The concomitant increased risks of ischemic stroke/systemic embolism, coronary ischemic events, and antithrombotic treatment-related bleeding makes it challenging to determine what antithrombotic strategies to use in elderly patients with CAD and AF [8, 9]. Oral antithrombotic strategies are comprised of antiplatelet therapy (APT) and oral anticoagulant (OAC) therapy. APT is regarded as the cornerstone for the treatment of SCAD and acute coronary syndrome (ACS) [10]. OAC is essential for the treatment of AF because it reduces the risk of ischemic stroke [11, 12]. In patients with AF and ACS or undergoing percutaneous coronary intervention (PCI), the current guidelines recommend the use of a short course (4–6 weeks) of triple therapy (dual antiplatelet therapy with aspirin and P2Y12 inhibitors plus an OAC) followed by dual therapy (P2Y12 inhibitor plus an oral OAC) for up to 12 months [13, 14]. Overall, the long-term use of oral anticoagulants (OACs) is recommended for patients with ACS and AF treated with medical therapy or PCI. Several observational and prospective registries have evaluated the optimal antithrombotic treatment for patients with SCAD and AF [15–18]. Although the current guidelines recommend monotherapy with an OAC or combined OAC with single antiplatelet therapy (SAPT) in patients with SCAD and AF, particularly in AF patients with SCAD for > 1 year after ACS or PCI, there is great uncertainty regarding this strategy [8, 9, 19, 20]. For elderly patients with a high prevalence of SCAD and AF, the choice of antithrombotic treatment in real-world clinical practice is difficult due to the complex situation of the increased risk of both ischemia and bleeding events in elderly individuals. To date, no consensus or recommendation has been made regarding antithrombotic treatment in elderly patients with SCAD and AF, and there are currently no randomized controlled or real-world studies to guide our decision-making in treating these elderly patients in clinical practice [21]. To understand the efficacy and safety of OACs in elderly patients, this single center-based cohort study aimed to analyze real-world antithrombotic strategies with OAC application as well as the efficacy and safety of OACs in elderly patients with SCAD and AF.

## Methods

### Patients

Patients aged ≥ 65 years diagnosed with CAD and AF were consecutively recruited from the cardiology department of Chinese PLA General Hospital from 2010 to 2017. Participants were included in the study if they had both SCAD and nonvalvular AF. SCAD includes stable angina, previous myopathy infarction and ischemic cardiomyopathy [22]. Nonvalvular atrial fibrillation refers to atrial fibrillation without mechanical valve prosthesis and rheumatic mitral stenosis [8, 23]. Subjects were excluded if they had a reversible cause of AF and a known contraindication to antithrombotic therapy or a life expectancy of less than 12 months. In addition to patients with AF, patients with other indications for OAC (e.g., mechanical heart valve, pulmonary embolism, and left ventricular mural thrombus) with follow-up less than 12 months or who were lost to follow-up were excluded. This study complied with the Declaration of Helsinki and was approved by the institutional ethics committee of Chinese PLA General Hospital, and all patients provided written informed consent.

### Outcomes and follow-ups

The primary efficacy clinical outcomes were Major adverse cardiac events (MACEs) and bleeding events. MACEs were defined as all-cause death, nonfatal myocardial infarction (MI), nonfatal stroke, and systemic embolism. Bleeding events were defined according to the Bleeding Academic Research Consortium (BARC) criteria. Major bleeding events were defined as events that were BARC ≥ 3, and clinical bleeding events were defined as events that were BARC ≥ 2. The net clinical outcomes included MACEs and BARC ≥ 3 events. The clinically important events, readmissions and drug treatment plans of the enrolled patients were collected through telephone follow-up. The enrolled patients were followed until death or until the end of the study (December 31, 2017).

### Statistical analysis

The CHA_2_DS_2_-VASc score and HAS-BLED score were used to standardize the risk of stroke or bleeding in the patients. The CHA_2_DS_2_-VASc score was calculated as congestive heart failure (1 point), hypertension (1 point), age ≥ 75 years (2 points), diabetes (1 point), stroke/transient ischemic attack/thromboembolism (2 points), vascular disease (prior myocardial infarction, peripheral artery disease, or aortic plaque: 1 point), age 65 to 74 years (1 point), and female sex (1 point). The modified HAS-BLED score was calculated as hypertension (1 point), abnormal renal and liver function (1 point each), stroke (1 point), bleeding (1 point), elderly (e.g., age > 65 years: 1 point), drugs or alcohol (1 point each). The SPSS 22.0 system was used for the statistical description and analysis. Continuous variables are expressed as the mean ± standard deviation (SD) or median and were compared using the t test or Mann‒Whitney U test based on their distributions. Categorical variables are expressed as the number and percentage and were compared using the χ^2^ test as appropriate. When *P* < 0.05, there was a significant difference. Multivariate logistic regression analysis was carried out for the characteristics that were significantly different between the groups to determine the independent predictive capability of OAC treatment on the clinical outcome. Kaplan–Meier estimates of MACEs and bleedings (BARC ≥ 3) were used to construct time-to-event curves. All tests were two-tailed, and *P* values < 0.05 were considered statistically significant.

## Results

### Patient characteristics

**Fig. 1:**
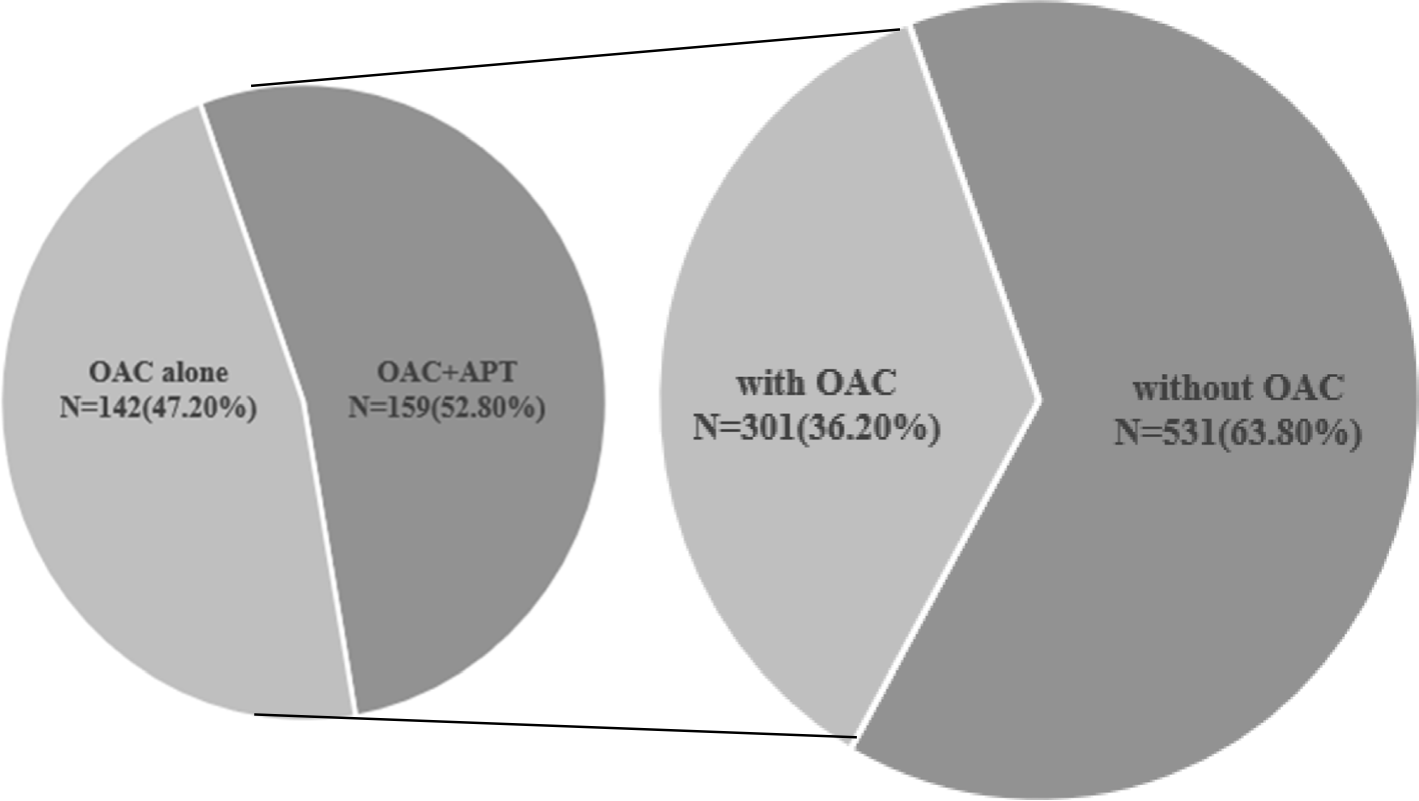
Prevalence of antithrombotic strategies. OAC: oral anticoagulant; APT: antiplatelet treatment; DOAC: new oral anticoagulant

**Fig. 2:**
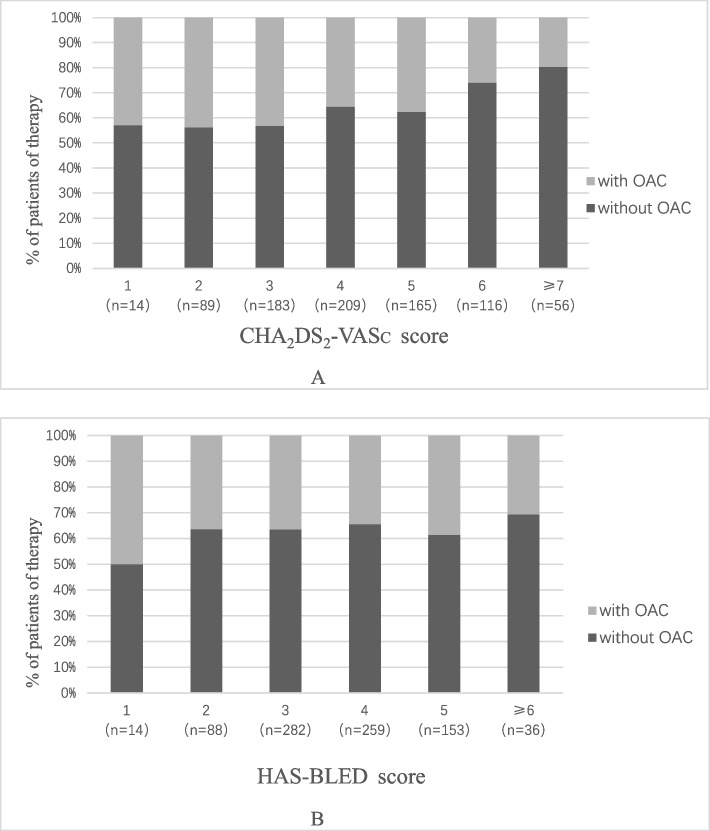
Distribution of antithrombotic strategies by CHA_2_DS_2_-VASc score (**A**) and HAS-BLED score (**B**)

Among the continuously enrolled 2,437 patients diagnosed with CAD and AF, and according to the inclusion and exclusion criteria, 832 elderly patients with SCAD and AF were finally included in the analysis. The baseline characteristics according to the different treatment plans (with or without OAC) are shown in Table 1. The mean age of the patients in the cohort was 78 years, and 351 patients were female. The compositions of the different antithrombotic regimens are shown in Fig. 1. There were 301 (36.18%) patients who received OAC therapy and 531 (63.82%) who did not receive OAC therapy. The patients with OAC were much younger, had a higher body mass index (BMI), had higher prevalence of statin administration, β-blocker administration, angiotensin receptor blocker (ARB), proton pump inhibitors (PPI) and APT, and they also had a lower prevalence of heart failure, chronic obstructive pulmonary disease, renal insufficiency, previous myocardial infarction and diuretic administration. No corresponding increase in the proportion of patients receiving OACs could be found with the increasing of CHA_2_DS_2_-VASc scores (Fig. 2A). Similarly, with the increasing of HAS-BLED score, the percentage of patients treated with OACs was not decrease significantly (Fig. 2B).

**Table 1:** Baseline clinical characteristics

Characteristics	Total (*n* = 832)	with OAC (*n* = 301)	without OAC (*n* = 531)	*P* value
Age (mean ± SD)	78 ± 6.7	77 ± 6.1	78 ± 6.9	0.000
Female (n, %)	351(42.2)	135(44.9)	216(40.7)	0.244
BMI (mean ± SD)	24.7 ± 3.8	25.2 ± 3.5	24.3 ± 3.9	0.001
**Comorbidity (n, %)**				
Hypertension	630(75.7)	228(75.7)	402(75.7)	1.000
Hyperlipidemia	171(20.6)	59(19.6)	112(21.1)	0.656
Diabetes	227(27.3)	80(26.6)	147(27.7)	0.747
Heart failure	307(36.9)	86(28.6)	221(41.6)	0.000
Chronic obstructive pulmonary disease	38(4.6)	6(2.0)	32(6.0)	0.009
Renal Insufficiency	121(14.5)	26(8.6)	95 (17.9)	0.000
Chronic Renal Insufficiency	95(11.4)	20(6.6)	75(14.1)	0.001
Malignant Tumor	116(13.9)	34(11.3)	82(15.4)	0.097
**Type of atrial fibrillation (n, %)**				
Paroxysmal	383(46.0)	128(42.5)	255(48.0)	0.129
Persistent	178(21.4)	75(24.9)	103(19.4)	0.065
Unclassified	271	98	173	
**History (n, %)**				
Previous myocardial infarction	108(13.0)	20(6.6)	88(16.6)	0.000
Previous stroke	211(25.4)	66(21.9)	145(27.3)	0.097
Previous bleeding	32(3.8)	13(4.3)	19(3.6)	0.580
**Concomitant medication (n, %)**				
Statins	633(76.1)	252(83.7)	381(71.8)	0.000
β-blockers	611(73.4)	238(79.1)	373(70.2)	0.006
ACEI	200(24.0)	62(20.6)	138(26.0)	0.091
ARB	349(41.9)	142(47.2)	207(39.0)	0.023
Diuretics	475(57.1)	146(48.5)	329(62.0)	0.000
Calcium Antagonists	448(53.8)	167(55.5)	281(52.9)	0.515
PPI	217(26.1)	143(47.5)	204(38.4)	0.013
APT	389(46.8)	159(52.8)	230(43.3)	0.009

*OAC* Oral anticoagulant, *SD* Standard deviation, *BMI* Body mass index, *ACEI* Angiotensin converting enzyme inhibitors, *ARB* Angiotensin receptor blocker, *PPI* Proton pump inhibitors, *APT* Antiplatelet treatment

### Efficacy and safety of OAC treatment in elderly patients with SCAD and AF

During the follow-up period, 375 (45.07%) patients had MACEs, including all-cause death in 287 patients, nonfatal MI in 3 patients, nonfatal stroke in 67 patients, and systemic embolism in 18 patients. The incidence of MACEs was significantly lower in the patients receiving OACs than in the patients not receiving OACs (20.60% vs. 58.95%, HR: 0.21, 95% CI: 0.15–0.30, *p* < 0.001) (Table 2).

**Table 2:** Risk of adverse clinical outcomes in patients with or without OAC treatment

Outcomes	with OAC (*n* = 301)	without OAC (*n* = 531)	Adjusted^a^ HR (95% CI)	*P* value
MACEs	62 (20.60)	313 (58.95)	0.21 (0.15–0.30)	0.000
All-cause death	29 (9.63)	258 (48.59)	0.12 (0.08–0.20)	0.000
Cardiac death	12(3.99)	65(12.24)	0.44(0.22–0.86)	0.016
Non-fatal MI	0 (0)	3 (0.56)	0 (0)	0.999
Non-fatal stroke	26 (8.64)	41 (7.72)	1.03 (0.61–1.77)	0.900
Systemic embolism	7 (2.33)	11 (2.07)	1.30 (0.46–3.63)	0.619
Bleedings	86 (28.57)	65 (12.24)	2.66 (1.81–3.91)	0.000
BARC ≥ 3	14 (4.65)	7 (1.32)	4.71 (1.75–12.64)	0.002
BARC ≥ 2	25 (8.31)	19 (3.58)	2.63 (1.36–5.08)	0.004
Net clinical outcomes^b^	70(23.26)	313(58.96)	0.27(0.19–0.38)	0.000

*MACEs* Major adverse cardiovascular events, including all-cause death, non-fatal MI, non-fatal stroke and systemic embolism, *OAC* Oral anticoagulant, *BARC* Bleeding Academic Research Consortium, *HR* Hazard ratio, *CI* Confidence interval

^a^For MACEs, HR was adjusted by the variables including sex, age, BMI, heart failure, renal insufficiency, chronic renal insufficiency, malignant tumor, chronic obstructive pulmonary disease, previous myocardial infarction, previous stroke, statins, β-blockers, angiotensin receptor blocker, diuretics, proton pump inhibitors, antiplatelet treatment. For bleeding events, HR was adjusted by the variables including sex, age, BMI, heart failure, renal insufficiency, chronic renal insufficiency, malignant tumor, chronic obstructive pulmonary disease, previous myocardial infarction, statins, β-blockers, angiotensin receptor blocker, diuretics, proton pump inhibitors, antiplatelet treatment, previous bleeding; For net clinical outcomes, HR was adjusted by the variables including sex, age, BMI, heart failure, renal insufficiency, chronic renal insufficiency, malignant tumor, chronic obstructive pulmonary disease, previous myocardial infarction, previous stroke, statins, β-blockers, angiotensin receptor blocker, diuretics, proton pump inhibitors, antiplatelet treatment, previous bleeding.

^b^Net clinical outcomes were defined as MACEs and BARC ≥ 3 type bleeding events

**Fig. 3:**
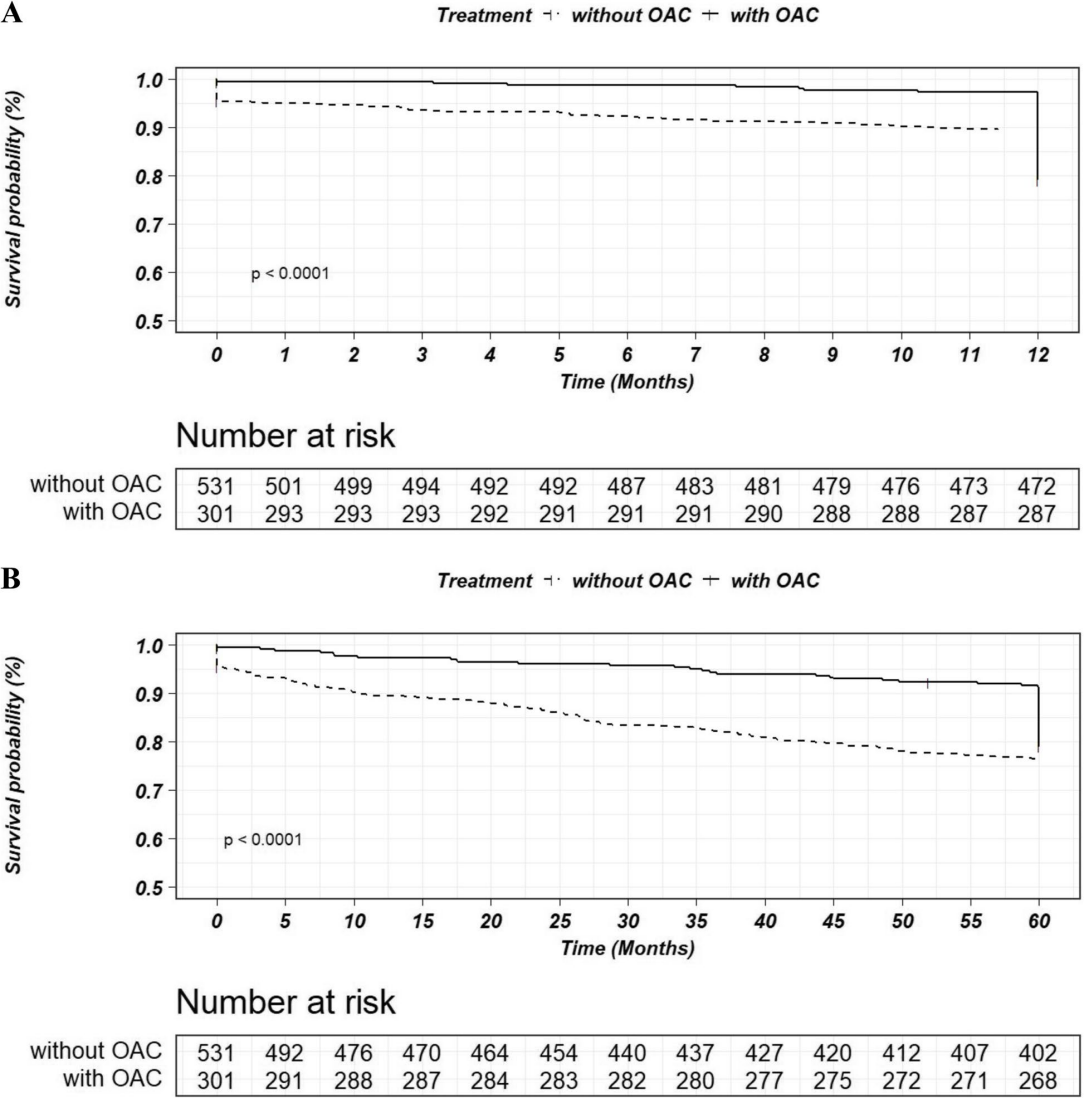
Kaplan–Meier survival curves for the endpoints of MACEs within a follow-up of 1 year (**A**) or 5 years (**B**) in patients with or without OAC. OAC: oral anticoagulant

**Fig. 4:**
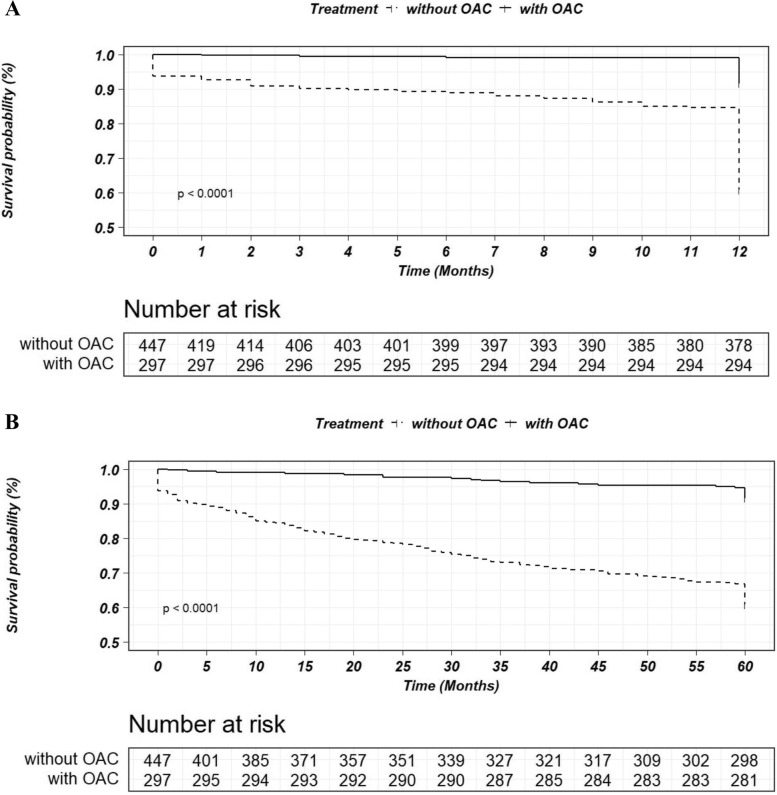
Kaplan–Meier survival curves for the endpoints of All-cause death within a follow-up of 1 year (**A**) or 5 years (**B**) in patients with or without OAC. OAC: oral anticoagulant

In terms of safety outcomes, bleeding events occurred in 151 (18.15%) patients, including major bleeding events (BARC ≥ 3) in 21 (2.52%) and clinically relevant bleeding events (BARC ≥ 2) in 44 (5.29%) patients. The incidence rate of bleeding events was significantly higher with OAC (28.57% vs. 12.24%, HR: 2.66, 95% CI: 1.81–3.91, *p* < 0.001) (Table 2). The incidence of net clinical outcomes was significantly lower in the patients treated with OACs than in those without OAC treatment (23.26% vs. 58.96%, HR: 0.27, 95% CI: 0.19–0.38, *p* < 0.001) (Table 2). Compared with the patients without OACs treatment, a significant decrease in MACEs as well as all-cause death could be found in patients treated with OACs within both 1-year and 5-year follow-ups (Figs. 3 and 4). However, no significant different for bleeding events (type BARC ≥ 3) could be observed between the groups (data not shown).

### Efficacy and safety of OAC + APT treatment in elderly patients with SCAD and AF

We also performed a subgroup analysis of the efficacy and safety of combined antiplatelet therapy in the OAC-treated patient group. Subgroup analyses were performed in 159 (52.82%) patients who were given both antiplatelet and OAC therapy and in 142 (47.18%) patients receiving anticoagulation alone. The characteristics of the patients on combination antiplatelet therapy were comparable to those of the patients on anticoagulation alone, except AF type and statins treatment percentage. After multivariable adjustment, the incidence of all-cause death was significantly lower in the patients treated with APT than in those without APT (5.66% vs. 14.08%, HR: 0.36, 95% CI: 0.16–0.85, *p* = 0.020) (Table 3). However, the incidence of nonfatal stroke was significantly higher in the patients treated with APT than in those who were not treated with APT (11.95% vs. 4.93%, HR: 3.46, 95% CI: 1.35–8.88, *p* = 0.010). In terms of safety outcomes, a higher incidence rate of bleeding events with APT treatment was found (32.70% vs. 23.94%, HR: 1.77, 95% CI: 1.04–3.03, *p* = 0.036) (Table 3). No significant difference in the net clinical outcomes could be found between the patients with and without APT (23.90% vs. 22.54%, HR: 1.30, 95% CI: 0.73–2.29, *p* = 0.372) (Table 3).

**Table 3:** Risk of adverse clinical outcomes in OAC treated patients with or without APT treatment

Outcomes	OAC with APT (No.of patients) (*n* = 159)	OAC without APT (No.of patients) (*n* = 142)	Adjusted^a^ HR (95% CI)	*P* value
MACEs	33 (20.75)	29 (20.42)	1.18 (0.66–2.12)	0.583
All-cause death	9(5.66)	20(14.08)	0.36(0.16–0.85)	0.020
Non-fatal MI	0(0)	0(0)	-	-
Non-fatal stroke	19(11.95)	7(4.93)	3.46(1.35–8.88)	0.010
Systemic embolism	5(3.14)	2(1.41)	2.64(0.46–15.33)	0.278
Bleedings	52(32.70)	34 (23.94)	1.77(1.04–3.03)	0.036
BARC ≥ 3	9(5.66)	5(3.52)	2.03(0.63–6.52)	0.235
BARC ≥ 2	18(11.32)	7(4.93)	2.71(1.07–6.90)	0.036
Net clinical outcomes^b^	38(23.90)	32(22.54)	1.30(0.73–2.29)	0.372

*MACEs* Major adverse cardiovascular events, including all-cause death, non-fatal MI, non-fatal stroke and systemic embolism, *OAC* Oral anticoagulant, *BARC* Bleeding Academic Research Consortium, *HR* Hazard ratio, *CI* Confidence interval

^a^For MACEs, HR was adjusted by the variables including sex, age, BMI, previous myocardial infarction, previous stroke, statins, persistent atrial fibrillation. For bleedings, HR was adjusted by the variables including sex, age, BMI, statins, persistent atrial fibrillation, previous bleeding; For net clinical outcomes, HR was adjusted by the variables including sex, age, BMI, statins, persistent atrial fibrillation, previous bleeding, previous myocardial infarction, and previous stroke;

^b^Including MACEs and BARC ≥ 3 type bleeding events

## Discussion

The main finding of the present study is that OAC treatment could significantly reduce the risk of MACEs but at the cost of an increased risk of major bleeding events in elderly patients with SCAD and AF. However, the net clinical benefit could still be observed in the OAC-treated patients with or without antiplatelet treatment. This finding indicates that for elderly patients with SCAD and AF, the greatest benefit of OAC treatment could be obtained in those with a high risk of ischemic cardiovascular events but with a low risk of bleeding. To the best of our knowledge, the present study is the first to provide real-world evidence for the individualization of OACs in elderly patients with SCAD and AF.

In our study, only 36.18% of the elderly patients with both SCAD and AF were treated with OACs. That proportion was comparable to the 44.7% that was recently reported in elderly Chinese patients with AF alone [24] and the 36.5% that was reported in Chinese AF patients with a CHA2DS2-VASc score ≥ 2 [25]. The reason for the underutilization of OACs in the present study could be attributed to many factors, such as age, type of AF, acute myocardial infarction (AMI), PCI, and the concomitant use of double antiplatelet therapy (DAPT).

Long-term therapy with OACs has been recommended in patients with CAD and AF [8]; however, appropriate antithrombotic treatment was less likely in elderly patients with CAD and AF [26], who tend to suffer more from ischemic adverse events [8, 27].

Based on the present real-world study, however, we found that the antithrombotic strategies in elderly patients with SCAD and AF were determined mainly not by the risk of cardiovascular ischemic event but by the risk of bleeding because, as this study showed, the proportion of OAC-treated patients did not increase with increasing CHA_2_DS_2_-VASc scores, while had a general tendency to decrease with increasing HAS-BLED scores. The similar situation was observed in elderly patients with ACS and AF [28]. Therefore, although the guidelines recommend the administration of OACs in patients with CAD and AF without the limitation of age, the actual situation is obviously that there is insufficient OAC use in the elderly maybe because of the high risk of bleeding. The effectiveness of oral OAC in elderly patients with SCAD and AF is reflected mainly by the more than 5 times absolute decrease in all-cause mortality, including an approximately 3 times decrease in cardiac death. A similar efficacy of OAC treatment was reported in elderly patients with AF [24, 29, 30].

However, OAC treatment conferred a higher risk of major bleeding in the present elderly cohort. Several reasons might account for the high risk of bleeding in OAC-treated patients. Among the present OAC-treated patients, a total of 63% were treated with direct oral anticoagulant (DOAC), and the remaining patients were treated with warfarin. Previous studies confirmed that in comparison to warfarin, direct oral anticoagulants (DOACs) are uniformly associated with an overall reduced risk of intracranial bleeding when used for stroke prevention in AF [31], especially in elderly individuals. Of the 301 included patients receiving OACs, 112 patients (37.21%) were treated with warfarin, and 189 (62.79%) were treated with DOACs. However, compared with DOACs, warfarin did not increase the total risk of major bleeding events.

Among the OAC-treated patients, 6 patients were observed to have intracranial bleeding, with 5 treated with warfarin and 1 treated with DOAC. Therefore, with the wide replacement of warfarin with DOAC in AF, the risk of intracranial bleeding could be extensively decreased in elderly individuals. Apart from intracranial bleeding, another main source of major bleeding in the OAC-treated patients was gastrointestinal bleeding (*n* = 7), with 5 treated with DOAC and 2 treated with warfarin. Strong evidence has confirmed that DOACs, including dabigatran, rivaroxaban, and edoxaban, are associated with a higher risk of gastrointestinal bleeding [32]. Due to the increased risk of gastrointestinal bleeding compared with warfarin and the reported bleeding rates with dabigatran and rivaroxaban when they are used for long-term treatment, the updated Beers criteria of the 2019 American Geriatrics Society have recommended caution in the use of DOACs for the treatment of venous thromboembolism or AF in adults 75 years or older [33]. Thus, DOACs should be prescribed with caution, especially among elderly patients with high-risk bleeding. Our results further illustrate the need for minimizing modifiable risk factors for gastrointestinal bleeding in elderly patients on DOACs. Despite the higher risk of major bleeding in OAC-treated patients, net clinical benefits could be observed in the OAC-treated elderly patients, suggesting that the perceived benefits outweighed the potential harms posed by the bleeding events.

Recent guidelines recommended that a short course of dual therapy with OAC and an antiplatelet agent (preferably P2Y12) should be considered as a preferred antithrombotic strategy in the therapeutic management of patients with both ACS and AF [8, 34]. However, no guidelines have been published for OACs in patients with SCAD and AF [8, 35]. Therefore, greater efforts to improve the administration of OACs in elderly individuals with SCAD and AF are necessary.

The efficacy and safety of OAC plus APT treatment among patients with SCAD and AF has been investigated, and an increase in bleeding events and a lower risk of ischemic events have been described [15, 16, 18, 21, 36]. The early termination of the OAC-ALONE study showed that there was no significant difference in the net clinical benefits and bleeding events between OAC alone and OAC plus APT [37]. The guidelines recommend that APT should be added to OAC for SCAD and AF patients with a high ischemic risk but not with a high bleeding risk [38]. Among the patients enrolled in the present study who received OAC treatment, 159 (52.82%) were treated with the antiplatelet agent combination. Although OAC plus APT could reduce the risk of all-cause mortality, the risk of clinically relevant bleeding events (BARC ≥ 2) increased. As a consequence, the net clinical benefit could not be obtained when OAC was combined with APT. Therefore, it is necessary to precisely evaluate the indications for the comedication of APT and OAC, especially in elderly individuals. Interestingly, we found that the risk of nonfatal stroke was higher in the patients who had treatment that included both OAC and APT. After tracing the stroke history of patients with nonfatal stroke, we found that the proportion of stroke history was much higher in the OAC with APT-treated patients (31.58% vs. 14.20%). This might partly be attributed to their higher prevalence of stroke as endpoints.

### Limitation

Several limitations of this study are worth considering. First, our observational real-world study demonstrated significantly different clinical outcomes between OAC and non-OAC therapy in patients with SCAD and AF. Due to the insufficient sample size, it was difficult to further classify and study the efficacy and safety of various anticoagulant drugs for treatment, resulting in insufficient research on the causes of the high bleeding risk. We did not calculate the average dose of anticoagulant and antiplatelet agent for each patient, due to the small sample size for subgroup analysis of dose titration of different type of medications. Second, the clinical follow-up data were all collected by specialized medical staff in the department of cardiology. Although the data were validated, some follow-up data may be biased by the memory of patients and their families, which might lead to a possible risk of recall bias. In addition, the study was based on a single center cohort, and the findings need to be further validated in large multicenter cohorts.

## Conclusion

The net clinical benefits of efficacy and safety could be observed in OAC-treated elderly patients with SCAD and AF. The benefit is independent of the comedications of oral antiplatelet treatment.

## Data Availability

The data used to support the findings of this study are available from the corresponding author (yintong301@163.com) upon request.
